# Morphological identification of ticks and molecular detection of tick-borne pathogens from bare-nosed wombats (*Vombatus ursinus*)

**DOI:** 10.1186/s13071-020-04565-6

**Published:** 2021-01-19

**Authors:** Danielle Beard, Hayley J. Stannard, Julie M. Old

**Affiliations:** 1 School of Science, Western Sydney University, Penrith, New South Wales Australia; 2 School of Animal and Veterinary Sciences, Charles Sturt University, Wagga Wagga, NSW Australia

**Keywords:** Wombat, Tick, Microbiome, Marsupial, 16S ribosomal RNA gene, Next-generation sequencing, Bacteria

## Abstract

**Background:**

Ticks are obligate haematophagous ectoparasites of vertebrate hosts and transmit the widest range of pathogenic organisms of any arthropod vector. Seven tick species are known to feed on bare-nosed wombats (*Vombatus ursinus*), in addition to the highly prevalent *Sarcoptes scabiei* mite which causes fatal sarcoptic mange in most bare-nosed wombat populations. Little is known about the pathogens carried by most wombat ticks or how they may impact wombats and wombat handlers.

**Methods:**

Wombat ticks were sourced from wildlife hospitals and sanctuaries across Australia and identified to species level using taxonomic keys. Genomic DNA was extracted from a subsample, and following the amplification of the bacterial 16S rRNA gene V3–V4 hypervariable region, next-generation sequencing (NGS) on the Illumina MiSeq platform was used to assess the microbial composition.

**Results:**

A total of 447 tick specimens were collected from 47 bare-nosed wombats between January 2019 and January 2020. Five species of ticks were identified comprising wombat tick *Bothriocroton auruginans* (*n* = 420), wallaby tick *Haemaphysalis bancrofti* (*n* = 8), bush tick *Haemaphysalis longicornis* (*n* = 3), common marsupial tick *Ixodes tasmani* (*n* = 12), and Australian paralysis tick *Ixodes holocyclus* (*n* = 4). Tick infestations ranged from one to 73 ticks per wombat. The wombat tick was the most prevalent tick species comprising 94% of the total number of samples and was present on 97.9% (46/47) of wombat hosts. NGS results revealed the 16S rRNA gene diversity profile was predominantly Proteobacteria (55.1%) followed by Firmicutes (21.9%) and Actinobacteria (18.4%). A species of *Coxiella* sharing closest sequence identity to *Coxiella burnetii* (99.07%), was detected in 72% of *B. auruginans* and a *Rickettsiella* endosymbiont dominated the bacterial profile for *I. tasmani*.

**Conclusions:**

A new host record for *H. longicornis* is the bare-nosed wombat. One adult male and two engorged adult female specimens were found on an adult male wombat from Coolagolite in New South Wales, and more specimens should be collected to confirm this host record. The most prevalent tick found on bare-nosed wombats was *B. auruginans*, confirming previous records. Analysis of alpha-diversity showed high variability across both sample locations and instars, similar to previous studies. The detection of various Proteobacteria in this study highlights the high bacterial diversity in native Australian ticks.
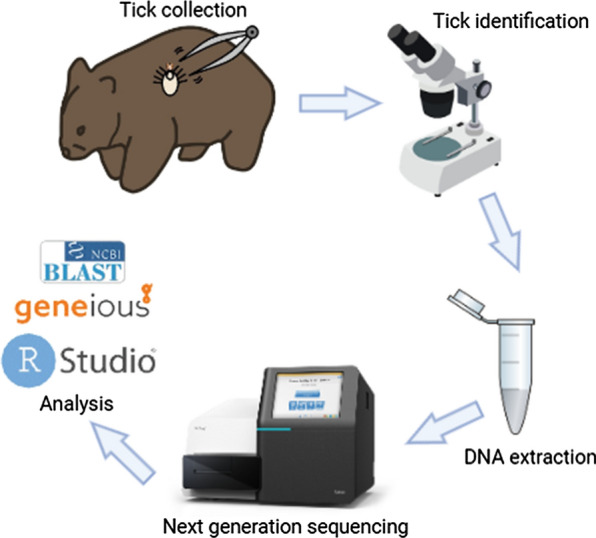

## Background

Ticks (Acari: Ixodidae) are obligate ectoparasitic arachnids that are classified into three families: Ixodidae (hard ticks), Argasidae (soft ticks), and Nuttalliellidae. Each of the three families have evolved unique biological, physiological and ecological disparities which have resulted in different abilities and capacities to transmit pathogens [1]. However, ticks can transmit the widest range of pathogens of any arthropod vector and are the primary cause of vector-borne diseases in livestock and domestic animals [2]. Ixodids transmit the widest number of pathogens worldwide and are responsible for the majority of tick-borne infections [3].

In addition to pathogens, the tick microbiome comprises a community of commensal and symbiotic obligate endosymbionts which make up the majority of the tick microbiome and reside both inside and outside the body of ticks [4]. The effect of these organisms has been somewhat neglected in studies, but may present various detrimental, neutral, or beneficial effects to their tick hosts, and also contribute to driving the transmission of tick-borne pathogens [5]. Non-pathogenic microorganisms are typically transovarially transmitted [6] and may impact tick growth, reproduction, fitness, nutritive adaptation and defence against environmental stresses [7, 8]. The functional roles of tick microorganisms and their relationships may provide further insights into the pathogenicity and evolution of tick pathogens. For example, it has become increasingly clear since the advancement of molecular barcoding techniques that many species of *Rickettsia*, *Francisella*, and *Coxiella*, which are generally considered pathogens of medical and veterinary importance, have evolved as non-pathogenic endosymbionts of ticks [9].

While tick-borne bacteria have been relatively well studied in the northern hemisphere, very little is known about the presence or diversity of bacteria in Australian ticks [10]. The microbiome and pathogenicity of Australian ticks are unique when compared to other species, and so is the response to ticks and tick-borne pathogens from native vertebrate hosts [11]. Recently, unique Australian species of *Anaplasma*, *Ehrlichia* and *Neoehrlichia* [12, 13] and the first native *Borrelia* species were characterised in native ticks [14]. Other novel microbial species have also been reported in Australian ticks [12, 15, 16]; however, the focus has largely been on ticks of human, domestic animal and livestock importance, and few studies have surveyed ticks associated with wildlife [17, 18].

Bare-nosed wombat (*Vombatus ursinus*) populations are significantly impacted by the ectoparasite *Sarcoptes scabiei*, which causes sarcoptic mange [19]; however, little is known about other wombat ectoparasites or their associated pathogens. Australian fauna have co-evolved with native tick species, and healthy wombats regularly carry large burdens of ticks which would otherwise affect humans and domestic animals [20]. However, wombats affected by sarcoptic mange, orphaned or injured wombats released from captivity and wombats raised in a comparatively parasite-free captive environment before release are likely at an increased risk of contracting tick-borne diseases. Managing wild species in captivity may induce stress, impair immunity and expose hosts to novel parasites to which the immune system is naïve [21]. Population density is also often atypical in captivity, which may result in higher than usual parasite burdens. Additionally, the use of anti-parasitic medications on captive animals affects both host-parasite relationships and individuals, as the latter are at an increased risk of disease once released, having not developed acquired immunity [22].

Seven species of ticks have previously been recorded feeding on bare-nosed wombats including the wombat tick *Bothriocroton auruginans* [23][23], wallaby tick *Haemaphysalis bancrofti* [24], Australian paralysis tick *Ixodes holocyclus* [25], Tasmanian paralysis tick *Ixodes cornuatus* [26], *Ixodes phascolomyis* [27], common marsupial tick *Ixodes tasmani* and *Ixodes victoriensis* [28][28]. The relationship between *S. scabiei* and other known wombat ectoparasites, their pathogens, ability to co-infect hosts, and their overall impact on wombat hosts have not yet been investigated. There is also very little known about the life cycles of wombat ectoparasites and their level of host specificity. *Coxiella burnetii* has been found in *B. auruginans* collected from bare-nosed wombats, as well as a *Rickettsia* species closely related to *Rickettsia massiliae*, which causes human disease [29]. These are the only pathogens that have been detected in ticks taken from wombat hosts, and were identified using specific targeted methods.

The development of next-generation sequencing (NGS) technologies has enabled the microbial communities of ticks to be explored in a fast and cost-efficient manner [15]; however, very little is known about the microbiome of native Australian ticks [10] and no studies have focused on wombat ticks or tick-borne pathogens. Bare-nosed wombats are already significantly affected by a known ectoparasite, so it would be beneficial to understand the other parasitic and pathogenic threats that wombats may need to overcome simultaneous to or following the treatment of sarcoptic mange. It is also important to identify potential zoonotic threats to wombat handlers and domestic animals that may come into contact with wombats or their burrows. This study aimed to identify the species of ticks associated with bare-nosed wombats and to use NGS and metabarcoding to investigate the bacterial diversity associated with these ticks.

## Methods

### Tick collection and identification

**Fig. 1:**
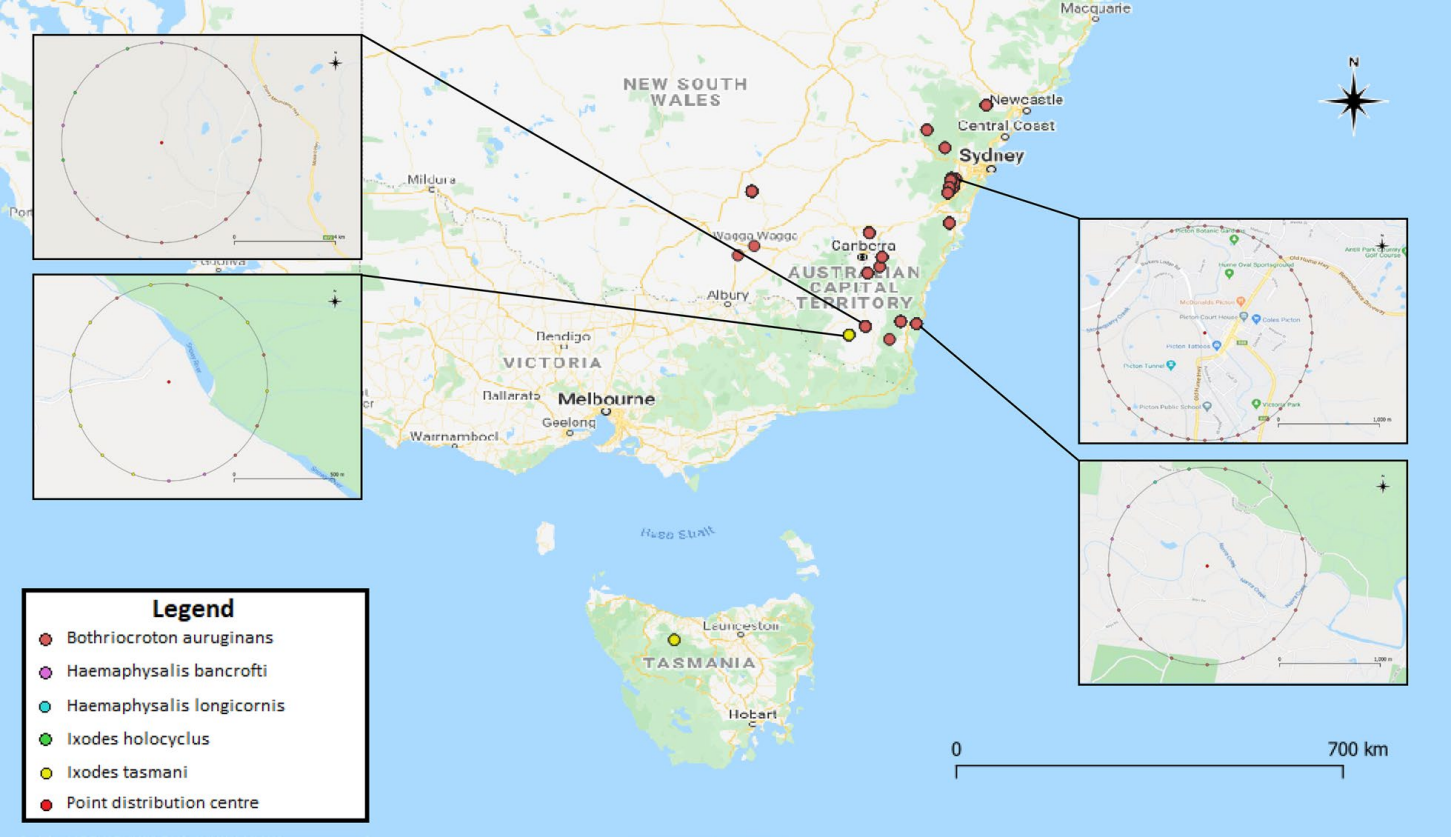
Geographic distribution of ticks collected from bare-nosed wombat (*Vombatus ursinus*) hosts between January 2019 and January 2020.* Each point* represents a unique collection location for the corresponding tick species. Overlapping points were displaced with a point displacement renderer around a centre symbol (denoted in the legend); point displacement distance was defined by number of map units (kilometres)

All ticks were collected directly from wombat hosts between January 2019 and January 2020 throughout eastern Australia (Fig. 1) including from live animals being rehabilitated for release, as well as opportunistic collections from road-killed wombats, and placed into 70% ethanol. The location where the ticks were collected, the date, and habitat type for the wombat hosts were recorded. Temperature and rainfall were obtained from the Bureau of Meteorology for the date and Global Positioning System coordinates where ticks were submitted. All ticks were identified morphologically to species and life stage using existing taxonomic keys [30, 31] and a Nikon SMZ445 stereomicroscope. Species, sex and instar were recorded for each specimen except for two nymphal specimens and specimens that were damaged during removal. There is a lack of detailed morphological keys for some Australian native ticks at the larval and nymphal stages [32], so some of these specimens could only be identified to genus level. Damaged ticks were identified to instar and genus. Photographs of tick specimens were taken using an Olympus DP72 stereomicroscope with an external Euromex EK-1 fibre optic light source and cellSens Standard version 1.5 software. Ticks were stored in sterile tubes containing 70% ethanol between identification and molecular analysis.

### Sample mapping

The locations of tick sample collection were geo-referenced using the open source software QGIS version 3.12.1 [33] with the latest Australian coordinate system Geocentric Datum of Australia 2020 incorporated through the ICSM NTv2 Transformer plugin [34]. Layers were styled with a categorised renderer and layer symbology was characterised according to tick species. To visualise overlapping points, a point displacement renderer was used around a centre symbol on rendering circles for tick distribution, and a point cluster renderer was used to visualise overlying pathogen distribution [35].

### Molecular methods

Samples were sent to the Australian Genome Research Facility in Urrbrae, Adelaide Australia. DNA was extracted using the DNeasy PowerSoil Pro DNA Extraction Kit (Qiagen, Venlo, the Netherlands) according to the manufacturer′s instructions. A total of 79 whole tick specimens were then sequenced on an Illumina MiSeq platform [36]. Based on previous studies [37], the presence of bacteria in tick samples was detected using the primer pair 341F (5′-CCTAYGGGRBGCASCAG-3′) and 806R (5′-GGACTACNNGGGTATCTAAT-3′) to amplify the V3-V4 region of the 16S rRNA gene, generating a 300-base pair fragment.

The bioinformatics analysis involved demultiplexing, quality control, operational taxonomic unit (OTU) clustering, and taxonomic classification. Image analysis was performed in real time using MiSeq Control Software version 2.6.2.1 and Real Time Analysis version 1.18.54 (Illumina, San Diego, CA), running on the instrument computer. Then the Illumina bcl2fastq 2.20.0.422 pipeline was used to generate the sequence data. Paired-ends reads were assembled by aligning the forward and reverse reads using PEAR version 0.9.5 [38], and primers were identified and trimmed. Trimmed sequences were processed using Quantitative Insights into Microbial Ecology (QIIME) version 1.8.4 [39], USEARCH version 8.0.1623 [40], and UPARSE [41] software. Using USEARCH tools, sequences were then quality filtered, full-length duplicates were removed and sequences were sorted by abundance. Singletons or unique reads were discarded, sequences were clustered and chimeric sequences were filtered using the rdp_gold database as a reference. To obtain the number of reads in each OTU, reads were mapped back to OTUs with a minimum identity of 97%, taxonomy was assigned using QIIME and taxonomies were confirmed using the National Center for Biotechnology Information MegaBLAST. Non-bacterial (eukaryote, unidentified) OTUs were removed and samples with <100 assigned OTUs were not considered a positive identification.

### Data management and statistical analyses

Tick collection and identification details were recorded in Microsoft Excel version 2002. Quality assurance was ensured prior to statistical analyses by reviewing all physical data and data entries. Statistical analyses and data visualization were performed using RCommander version 2.6-2 [42], RStudio version 1.2.5033 [43] with the addition of packages vegan version 2.5-6 [44] and phyloseq [45], and Geneious Prime 2020.1.1 (https://www.geneious.com). Alpha-diversity was assessed by richness (inverse Simpson and ACE index) and diversity (Shannon and Simpson index).

## Results

### Tick species

**Fig. 2:**
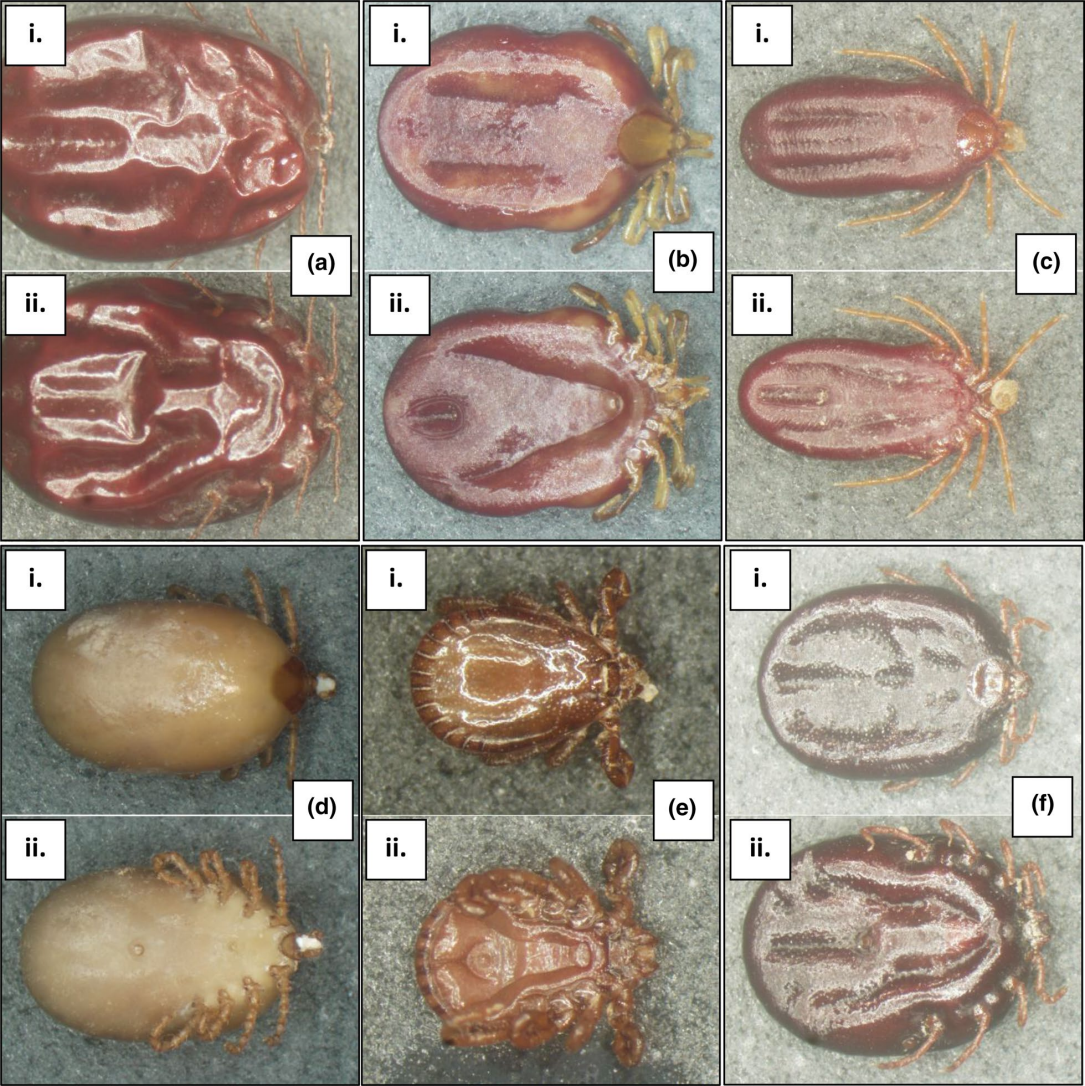
**a** Bush tick *Haemaphysalis longicornis* female (i) dorsal, (ii) ventral; **b** Australian paralysis tick *Ixodes holocyclus* female (i) dorsal, (ii) ventral; **c** common marsupial tick *Ixodes tasmani* female (i) dorsal, (ii) ventral; **d** wombat tick *Bothriocroton auruginans* female (i) dorsal, (ii) ventral; **e** bush tick *Haemaphysalis longicornis* male (i) dorsal, (ii) ventral; **f** wallaby tick *Haemaphysalis bancrofti* female (i) dorsal, (ii) ventral

**Fig. 3:**
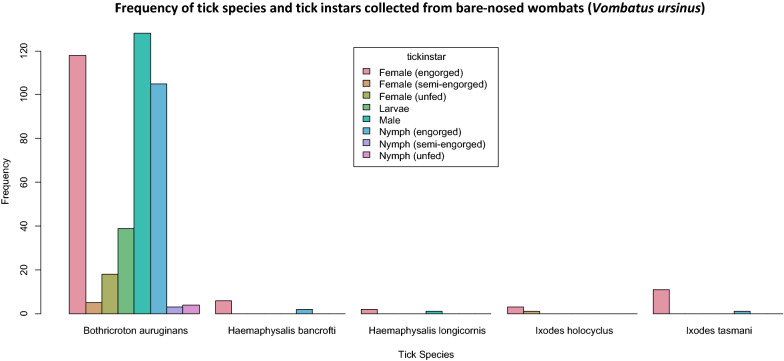
Species of tick and frequency of each instar collected from bare-nosed wombat (*V. ursinus*) hosts between January 2019 and January 2020

A total of 447 tick specimens were collected from 47 bare-nosed wombats in New South Wales (NSW) and Tasmania between January 2019 and January 2020 (Table 1). Five species of ticks comprising three genera were morphologically identified (Table 2); wombat tick (*n* = 420; Fig. 2d), wallaby tick (*n* = 8; Fig. 2f), bush tick (*n* = 3; Fig. 2a, e), Australian paralysis tick (*n* = 4; Fig. 2b), and *I. tasmani* (*n* = 12; Fig. 2c). Approximate tick infestation ranged from one to 73 ticks per wombat with a total mean infestation of 9.8 ± 3.9 ticks per host. Juvenile (joey at foot) wombats exhibited higher mean infestation rates (25.3 ± 20.4), followed by adult female wombats (7.1 ± 4.5) and adult male wombats (6.6 ± 3.6). The wombat tick was the most prevalent tick species comprising 94% of the total number of samples and was present on 97.9% (46/47) of wombat hosts. Approximate tick diversity ranged from one to four tick species per wombat. The highest tick diversity was from an adult male wombat in Coolagolite in NSW, an adult male wombat from Dalgety NSW and a wombat of unknown age and sex at Quaama NSW with three tick species identified for each. Females were the most abundant instar identified (*n* = 164), followed by males (*n* = 129), nymphs (*n* = 115), and larvae (*n* = 39). The majority of females (89%), nymphs (96.5%) and larvae (100%) were engorged or semi-engorged from a blood meal (Fig. 3). Larvae could be identified to genus level only. In addition to ticks, there were also incidental collections of nine unidentified fleas and six lice (all of the latter were identified as *Boopia tarsata*). Most ticks were collected in winter (58%), followed by spring (25%), autumn (6%) and summer (6%); the remaining ticks were older specimens for which only the year of collection was recorded.

**Table 1:** Study population of bare-nosed wombats (*Vombatus ursinus*) used for tick collection in this study

Collection location	GPS coordinates	No. of hosts	No. of ticks
Cedar Creek, NSW	32°49′30.32″S, 151°9′2.23″E	7 (6 ♂, 1 ♀)	32 (17 ♂, 15 ♀)
Rock Flat, NSW	36°25′34.0284″S, 149°11′2.7132″E	2 (1 ♂, 1 U)	18 (2 N, 16 ♀)
Bells Line of Road, NSW	33°31′1.0272″S, 150°28′47.316″E	1 ♀	5 (2 ♂, 3 ♀)
Murrabrine Forest Road, Yowrie, NSW	36°20′42.576″S, 149°45′33.12″E	2 ♂	9 (1 N, 1 ♂, 7 ♀)
Bridge over Colombo Creek, Bemboka, NSW	36°38′8.9052″S, 149°34′38.1792″E	1 ♀	2 ♀
Rilys Road, Coolagolite, NSW	36°22′58.6416″S, 150°0′53.91″E	2 (1 ♂, 1 ♀)	4 ♀
Wolgan Valley, NSW	33°13′42.978″S, 150°11′10.2948″E	2 U	6 (1 L, 5 N)
Wagga Wagga, NSW	35°6′54.6696″S, 147°22′32.5344″E	1 ♂	1 ♂
The Rock, NSW	35°16′5.1528″S, 147°6′43.668″E	1 U	1 ♂
Werombi Road, Orangeville, NSW	34°1′23.8728″S, 150°39′22.7088″E	1 U	6 (4 N, 2 ♀)
West Parade, Thirlmere, NSW	34°13′16.9932″S, 150°33′26.55″E	1 U	10 (4 N, 3 ♂, 3 ♀)
West Parade, Couridjah, NSW	34°13′38.6472″S, 150°33′11.124″E	1 U	9 (7 N, 2 ♀)
Picton, NSW	34°10′9.2856″S, 150°36′32.5008″E	1 U	41 (28 N, 7 ♂, 6 ♀)
Spring Creek Road, Mount Hunter, NSW	34°4′53.976″S, 150°37′46.2108″E	1 ♂	5 (4 ♂, 1 ♀)
Eastview Drive, Orangeville NSW	34°0′54.0756″S, 150°35′11.9508″E	1 ♂ (J)	16 (6 ♂, 10 ♀)
Silverdale Road, The Oaks, NSW	34°4′8.1624″S, 150°34′25.6656″E	1 ♀	10 (5 ♂, 5 ♀)
Moulders Road, Orangeville, NSW	34°2′44.4804″S, 150°34′23.4732″E	1 ♀	11 ♀
Couridjah, NSW	34°13′54.8832″S, 150°32′58.0308″E	1 ♀	9 (2 ♂, 7 ♀)
Pheasants Nest Road, Pheasant Nest, NSW	34°15′15.318″S, 150°37′47.9784″E	1 ♂ (P)	2 N
Mowbray Park Road, Mowbray Park, NSW	34°9′39.51″S, 150°32′54.1428″E	1 ♂	4 (1 ♂, 3 ♀)
Buxton Road, Buxton, NSW	34°15′4.5108″S, 150°31′34.1688″E	1 ♀	26 (2 N, 21 ♂, 3 ♀)
Kangaroo Valley, NSW	34°44′31.7436″S, 150°33′8.028″E	1 U (J)	73 (32 N, 15 ♂, 26 ♀)
Bellmount Forest, NSW	34°54′14.4612″S, 149°14′54.0888″E	1 ♀	4 (1 ♂, 3 N)
Bellmount Forest, NSW	34°53′58.7832″S, 149°14′53.2392″E	1 ♂	20 (3 N, 15 ♂, 2 ♀)
Holbrook Road, Gelston Park, NSW	34°13′33.9996″S, 147°20′14.3088″E	1 ♂	37 ♂
Rilys Road, Coolagolite, NSW	36°22′58.7244″S, 150°0′54.162″E	2 ♂	13 (3 N, 1 ♂, 9 ♀)
Captains Flat Road, Primrose Valley, NSW	35°27′14.8644″S, 149°25′8.0544″E	1 ♂	3 (1 ♂, 2 ♀)
Hard Road, Burra, NSW	35°33′28.4436″S, 149°13′19.3296″E	1 ♀ (J)	44 (38 L, 5 N, 1 ♂)
Ironmungie Road, Dalgety NSW	36°33′53.6148″S, 148°55′7.5288″E	1 ♂	7 ♀
Gidleigh Lane, Bungendore, NSW	35°17′43.656″S, 149°27′21.3192″E	1 ♀ (J)	14 N
U	U	1 U	10 (2 N, 8 ♀)
Cradle Mountain Road, Cradle Mountain, Tasmania	41°31′23.2716″S, 146°4′32.6388″E	1 ♀ (J)	3 (1 N, 2 ♀)

*NSW* New South Wales, *GPS* Global Positioning System, *U* unknown, *L* larvae, *N* nymph, *P* pinky (unfurred joey), *J* joey

**Table 2:** List of the tick species collected and identified from bare-nosed wombat (*V. ursinus*) hosts between January 2019 and January 2020

Tick species	Common name	No. collected	Instar	Locality
*Bothriocroton auruginans*	Wombat tick	420	128 ♂, 141 ♀, 112 N, 39 L	NSW: Coolagolite, Rock Flat, Yowrie, Bellmount Forest, Bilpin, Bemboka, Buxton, Primrose Valley, Courijah, Orangeville, Bungendore, Burra, Gelstone Park, Dalgety, Kangaroo Valley, Mowbray Park, Pheasant Nest, Picton, Quaama, The Oaks, Mount Hunter, The Rock, Wagga Wagga, Thirlmere, Wolgan Valley
*Haemaphysalis bancrofti*	Wallaby tick	8	8 ♀, 2 N	NSW: Coolagolite, Dalgety, Picton, Quaama
*Haemaphysalis longicornis*	Bush tick	3	1 ♂, 2 ♀	NSW: Coolagolite
*Ixodes tasmani*	Common marsupial tick	12	11 ♀, 1 N	NSW: Dalgety. Tasmania: Cradle Mountain
*Ixodes holocyclus*	Australian paralysis tick	4	4 ♀	NSW: Coolagolite, Quaama

For abbreviations, see Table 1

### NGS analysis and bacterial composition of wombat ticks

**Fig. 4:**
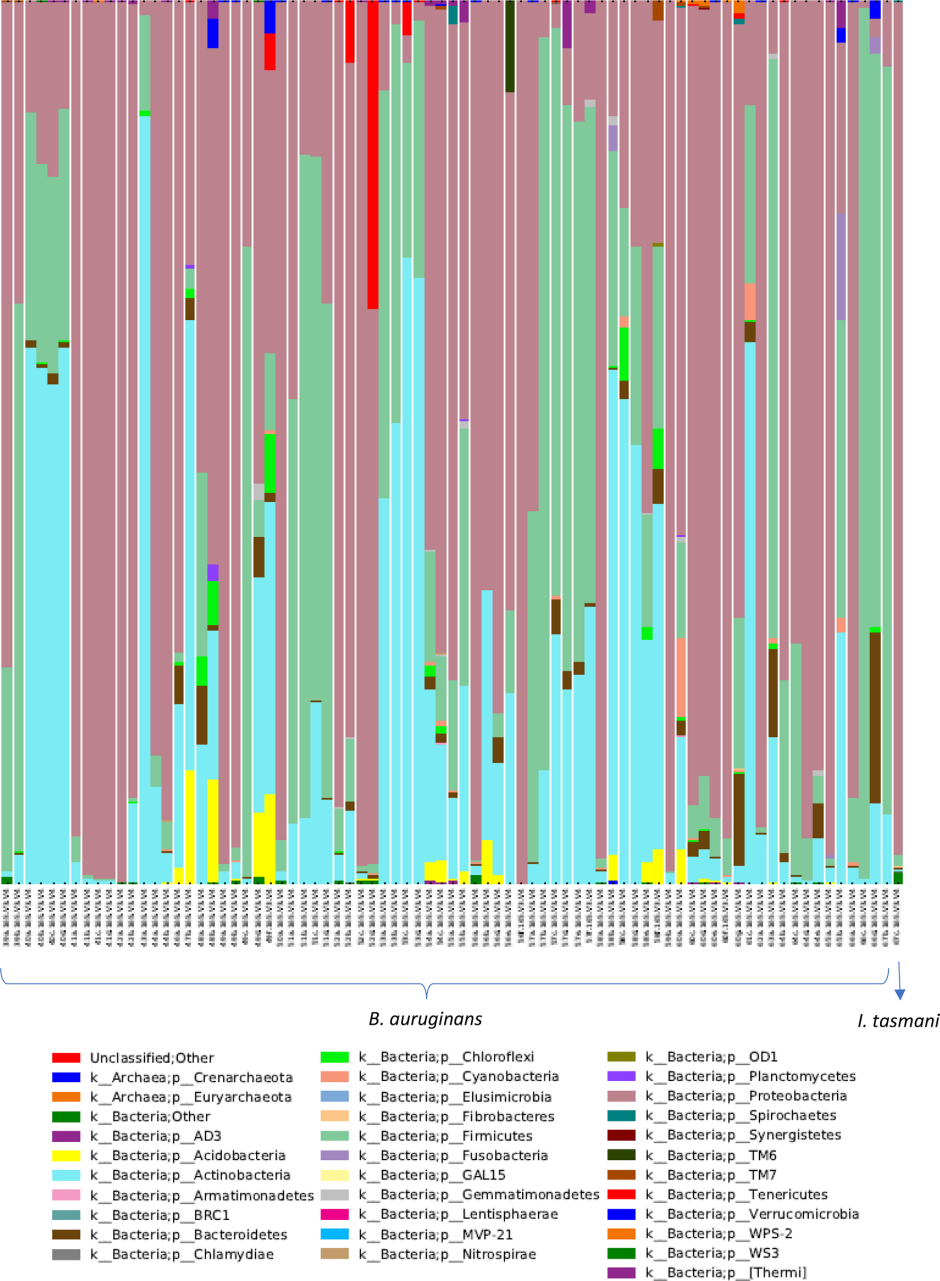
Taxonomic summary of bacterial phyla found in wombat ticks between January 2019 and January 2020

A total of 5,890,950 bacterial sequences and 1,759 OTUs (average length 414.3 bases) were assigned; however, only 745 OTUs had greater than 100 total sequences from all tick samples. Ticks had an average of 74,569 assigned sequences each (males 63,397 sequences, females 92,827 sequences, nymphs 56,470 sequences and larvae 57,701 sequences). Engorged females had an average of 99,550 assigned sequences in comparison to unfed females, which had an average of 40,723 sequences. The closest matches for bacterial isolates as determined through GenBank for taxa of interest are shown in Table 3. Proteobacteria comprised the majority of the bacterial phyla composition (55.1%) followed by Firmicutes (21.9%) and Actinobacteria (18.4%), as shown in Fig. 4. At the genus level *Coxiella* comprised 40.3% of the total composition followed by *Staphylococcus* (13%). *Coxiella* was the most dominant genus detected in larvae with a mean prevalence of 81.6%. Nymphs were less likely to be infected with one dominant phyla of bacteria than other instars and often exhibited equal frequencies of three phyla. Male and female adult ticks were predominantly associated with Proteobacteria (Table 4).

**Table 3:** Bacterial composition of ticks parasitising bare-nosed wombat (*V. ursinus*) hosts between January 2019 and January 2020

Tick species	Locality	Closest match in GenBank (% similarity)	No. positive	Length (bp)	Bit-score
*B. auruginans *(wombat tick)	NSW	*Coxiella burnetii* (99.07%)	56/78	429	771
		*Staphylococcus sciuri* (100%)	16/78	429	793
		*Corynebacterium amycolatum* (100%)	9/78	410	758
		*Dermacoccus nishinomiyaensis* (97.80%)	12/78	409	706
		*Macrococcus brunensis* (100%)	20/78	429	793
		*Planomicrobium glaciei* (100%)	4/78	428	791
		*Lysinibacillus* sp*.* (100%)	8/78	426	787
		*Brachybacterium paraconglomeratum* (100%)	13/78	409	756
		*Escherichia coli* (100%)	16/78	429	793
		*Acinetobacter* sp. (100%)	19/78	430	795
		*Pseudomonas* sp. (100%)	7/78	429	793
		Candidatus *Borrelia ivorensis* (99.53)	1/78	424	784
		Uncultured *Anaplasma* sp. (98.51%)	4/78	404	713
*I. tasmani *(common marsupial tick)	NSW	*Rickettsiella* endosymbiont (100%)	1/1	429	793

Only taxa of interest are shown, and numbers of positive samples are based on samples with > 100 assigned operational taxonomic units (OTUs)

*bp* Base pair

**Table 4:** List of wombat tick samples sequenced on the Illumina MiSeq platform and absolute OTU counts for each sample

Sample code	Species	Common name	Sex	Instar	Total abundance *C. burnetii*	Total abundance *Staphylococcus agnetis*	Total abundance *Rickettsiella*
39a	*B. auruginans*	Wombat tick	Female	Adult	96,850	240	0
39b	*B. auruginans*	Wombat tick	Female	Adult	38,832	617	0
40a	*B. auruginans*	Wombat tick	Male	Adult	424	86	0
40b	*B. auruginans*	Wombat tick	Male	Adult	499	37	0
40c	*B. auruginans*	Wombat tick	Male	Adult	0	52	0
40d	*B. auruginans*	Wombat tick	Male	Adult	245	33	0
41a	*B. auruginans*	Wombat tick	Female	Adult	118,871	23	0
41b	*B. auruginans*	Wombat tick	Female	Adult	135,810	1	0
41c	*B. auruginans*	Wombat tick	Female	Adult	151,567	0	0
41d	*B. auruginans*	Wombat tick	Female	Adult	35,440	0	0
42a	*B. auruginans*	Wombat tick	Female	Adult	121,194	0	0
42b	*B. auruginans*	Wombat tick	Male	Adult	11,701	0	0
43a	*B. auruginans*	Wombat tick	Female	Adult	42	49	0
44a	*B. auruginans*	Wombat tick	Female	Adult	22,187	3	0
44b	*B. auruginans*	Wombat tick	Female	Adult	150,092	74	0
46a	*B. auruginans*	Wombat tick	Female	Adult	8363	0	0
47b	*B. auruginans*	Wombat tick	Female	Adult	0	0	0
48a	*B. auruginans*	Wombat tick	-	Nymph	7320	0	0
48b	*B. auruginans*	Wombat tick	Female	Adult	694	0	0
49a	*B. auruginans*	Wombat tick	-	Nymph	56,494	0	0
49b	*B. auruginans*	Wombat tick	Male	Adult	82,607	155	0
49c	*B. auruginans*	Wombat tick	Male	Adult	28,893	51,956	0
49d	*B. auruginans*	Wombat tick	-	Nymph	0	0	0
49f	*B. auruginans*	Wombat tick	Male	Adult	0	1158	0
50a	*B. auruginans*	Wombat tick	Female	Adult	87,091	160	0
51a	*B. auruginans*	Wombat tick	Female	Adult	6965	328	54
51b	*B. auruginans*	Wombat tick	Female	Adult	172	100	0
51c	*B. auruginans*	Wombat tick	Male	Adult	0	194	0
51d	*B. auruginans*	Wombat tick	Male	Adult	0	293	69
52a	*B. auruginans*	Wombat tick	Female	Adult	47,707	1719	0
52b	*B. auruginans*	Wombat tick	Female	Adult	36,035	1682	0
52c	*B. auruginans*	Wombat tick	Male	Adult	107,270	195	0
52d	*B. auruginans*	Wombat tick	Male	Adult	47,213	432	0
53a	*B. auruginans*	Wombat tick	Female	Adult	12,669	520	0
53b	*B. auruginans*	Wombat tick	Female	Adult	3819	1029	0
53c	*B. auruginans*	Wombat tick	Female	Adult	2535	1130	0
53d	*B. auruginans*	Wombat tick	Female	Adult	2314	3475	0
54a	*B. auruginans*	Wombat tick	Female	Adult	47	2612	0
54c	*B. auruginans*	Wombat tick	Female	Adult	6442	137	0
55a	*B. auruginans*	Wombat tick	Female	Adult	76,233	6706	0
55b	*B. auruginans*	Wombat tick	Male	Adult	4771	119	0
56a	*B. auruginans*	Wombat tick	Male	Adult	72,344	0	0
56b	*B. auruginans*	Wombat tick	Male	Adult	0	0	0
56d	*B. auruginans*	Wombat tick	Male	Adult	20,493	0	0
56e	*B. auruginans*	Wombat tick	Male	Adult	2468	0	0
56f	*B. auruginans*	Wombat tick	Male	Adult	0	0	0
57a	*B. auruginans*	Wombat tick	-	Nymph	0	39,411	0
57b	*B. auruginans*	Wombat tick	-	Nymph	2	106,880	0
57c	*B. auruginans*	Wombat tick	-	Nymph	0	5956	0
57d	*B. auruginans*	Wombat tick	-	Nymph	0	5700	0
57e	*B. auruginans*	Wombat tick	-	Nymph	0	12,343	0
57f	*B. auruginans*	Wombat tick	-	Nymph	0	9720	0
58a	*B. auruginans*	Wombat tick	Male	Adult	107,406	44	0
58b	*B. auruginans*	Wombat tick	Male	Adult	1859	348	0
58c	*B. auruginans*	Wombat tick	Male	Adult	9	0	0
58d	*B. auruginans*	Wombat tick	-	Nymph	449	0	0
58e	*B. auruginans*	Wombat tick	-	Nymph	9918	0	0
58f	*B. auruginans*	Wombat tick	-	Nymph	8	0	0
59b	*B. auruginans*	Wombat tick	-	Nymph	93,668	0	0
60a	*B. auruginans*	Wombat tick	Male	Adult	8398	1543	0
60c	*B. auruginans*	Wombat tick	Male	Adult	128,271	30	0
60d	*B. auruginans*	Wombat tick	Male	Adult	92,622	99	0
60e	*B. auruginans*	Wombat tick	Male	Adult	99,967	3503	0
60f	*B. auruginans*	Wombat tick	Male	Adult	129,252	5470	0
60g	*B. auruginans*	Wombat tick	Male	Adult	55,074	903	0
61c	*B. auruginans*	Wombat tick	-	Nymph	1709	1	0
62a	*B. auruginans*	Wombat tick	Female	Adult	131,882	1	0
63a	*B. auruginans*	Wombat tick	Male	Adult	101	3536	0
64b	*B. auruginans*	Wombat tick	-	Larvae	37,394	1684	0
64c	*B. auruginans*	Wombat tick	-	Larvae	61,504	14,953	0
64d	*B. auruginans*	Wombat tick	-	Larvae	61,897	466	0
64e	*B. auruginans*	Wombat tick	-	Larvae	27,290	0	0
65a	*B. auruginans*	Wombat tick	Female	Adult	147,715	143	14
65b	*B. auruginans*	Wombat tick	Female	Adult	0	1453	1
66a	*B. auruginans*	Wombat tick	-	Nymph	118,704	969	0
66c	*B. auruginans*	Wombat tick	-	Nymph	0	17,181	0
66d	*B. auruginans*	Wombat tick	-	Nymph	0	3741	0
67b	*B. auruginans*	Wombat tick	-	Nymph	0	6881	0
67c	*Ixodes tasmani*	Common marsupial tick	Female	Adult	0	173	85,653

**Fig. 5:**
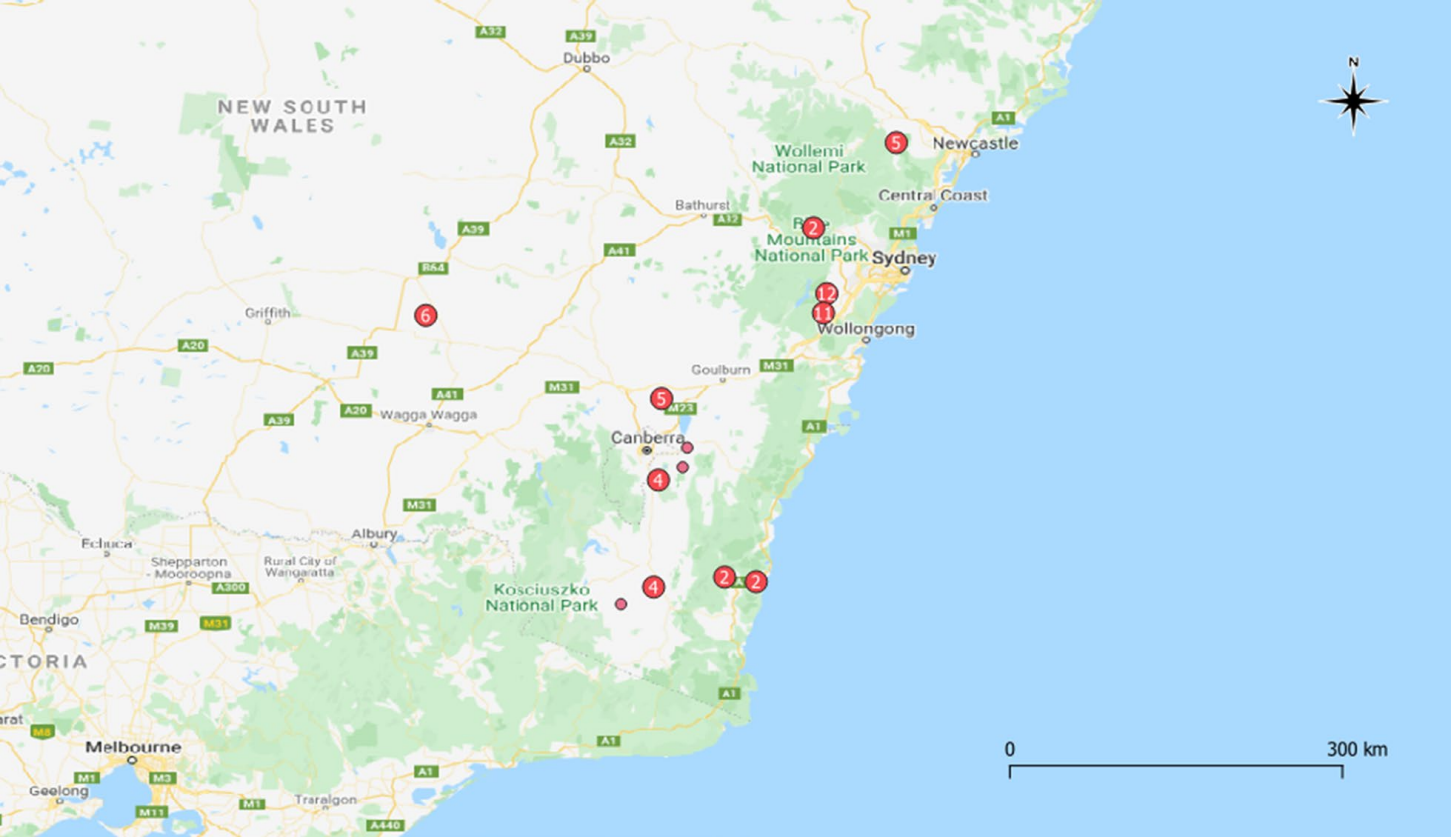
Geographic distribution of *Coxiella burnetii* detected in ticks from bare-nosed wombat (*V. ursinus*) hosts between January 2019 and January 2020.* Each point* corresponds with the collection location of the tick(s) which were positive (> 100 sequences) for *C. burnetii*. A point cluster renderer was used to group nearby points into a single rendered marker symbol. Point cluster distance was determined by point units

Four OTUs (OTU_1, LC464975, 99% identity; OTU_977, LC464975, 94.41% identity; OTU_1383, LC464975, 98.51% identity; and OTU_1806, CP014561, 93.26% identity) were identified as a species of *Coxiella* closest matched to *Coxiella burnetii* and were detected in 72% of *B. auruginans* (86% of females, 68% of males, 39% of nymphs and 100% of larvae) but not detected in *I. tasmani*. Females had a mean prevalence of 51.7%, males 30.7%, nymphs 19.6% and larvae 82.3% for *C. burnetii*. The distribution of *C. burnetii*-infected ticks detected in this study is shown in Fig. 5.

OTU_9 was assigned to a *Rickettsiella* endosymbiont of the common marsupial tick (KP994859, 100% identity) and comprised 94.5% of the bacterial diversity in the single female *I. tasmani* sample. This tick was collected from a wombat in Dalgety NSW, which is 100 km from the collection location of the wombat in Coolangubra NSW from which this sequence was originally isolated [6]. OTU_79 was assigned to Candidatus *Borrelia ivorensis* (KT364340, 99.53% identity) and was detected in only one engorged adult female *B. auruginans* (2051 sequences) from Mowbray Park in NSW. An uncultured *Anaplasma* sp. (OTU_29, MK041546, 98.51% identity) was detected in four female *B. auruginans* from Quaama, Coolagolite and The Oaks NSW.

The genus *Staphylococcus* was identified in six OTUs and was present in 66% of samples. OTU_14 (MT214233, 100% identity) was assigned to *Staphylococcus sciuri* and was present in 21% of *B. auruginans* samples (29% of females, 27% of males, no nymph or larvae) but not in *I. tasmani*. OTU_2 (MN314593, 100% identity), OTU_1817 (MN314593, 96.50% identity) and OTU_1923 (MN314593, 94.87% identity) had a top BLAST hit of *Staphylococcus agnetis* and were present in 56% of samples (60% of females, 41% of males, 56% of nymphs and 75% of larvae) including the common marsupial tick. Two additional OTUs were assigned to miscellaneous *Staphylococcus* spp. (OTU_15, MH549514, 100% identity; and OTU_1791, MG572712, 99.53% identity) but were represented in very low numbers of sequences.

Eight OTUs were assigned to the genus *Streptococcus*; however, only three were present in more than 100 sequences in any of the ticks. *Streptococcus dysgalactiae* (OTU_5, CP044102, 100% identity) was detected in very high sequence numbers in four female *B. auruginans* collected in Orangeville NSW. Nine *B. auruginans* (two females, three males, three nymphs and one larvae) had *Streptococcus salivarius* (OTU_51, MN559932 100% identity), and *Streptococcus didelphis* (OTU_1504, NR_115730, 99.53% identity) was detected in a low number of sequences (< 200) in one female and one nymph of *B. auruginans*.

*Escherichia coli* (OTU_4, NZ_CP045277, 100% identity) was identified in 21% of *B. auruginans* ticks (14% of females, 50% of nymphs, 50% of larvae and no males) but not in *I. tasmani*. OTUs that had a taxonomic identity associated with environmental bacteria such as Acidobacteria, Bacteroidetes and Cyanobacteria comprised <4% of the total composition. Skin and soil-associated bacteria that occurred in high sequence numbers included *Corynebacterium ulcerans* (OTU_20, 100% identity), *Corynebacterium amycolatum* (OTU_6, MK465377, 100% identity), *Macrococcus brunensis* (OTU_8, MK097326, 100% identity), *Comamonas serinivorans* (OTU_11, 9 CP021455, 9.77% identity), *Paraburkholderia caffeinilytica* (OTU_17, MN150516, 100% identity), and *Dietzia timorensis* (OTU_43, MN511783 100% identity).

## Discussion

This study aimed to record the species of ticks that feed on bare-nosed wombats and identify the bacteria associated with them. Five tick species were collected and included the first record of *H. longicornis* on bare-nosed wombats. A very high number of bacterial sequences were detected in wombat ticks, highlighting the effectiveness of NGS and the diversity of microorganisms in Australian ticks. Proteobacteria, Firmicutes and Actinobacteria dominated the bacterial profile, and the bacterial composition of the ticks studied supports similar investigations into these species [14, 17, 29].

The wombat tick *B. auruginans* is consistently the most prevalent tick found on bare-nosed wombats [46–48], and all instars except larvae were represented in this study. All larval specimens collected were identified as *Bothriocroton* sp. and shared their host with only *B. auruginans* instars. It is likely that these larval specimens were *B. auruginans* due to host specificity of other *Bothriocroton* spp.; however, this could not be confirmed. Heavy tick infestation has been associated with anaemia and poorer health parameters in other native marsupials [49, 50], and at least two of the wombats in this study were diagnosed with anaemia as a result of their tick burden (D. Kerr, personal communication). While it has been suggested that *B. auruginans* occurs throughout most of the bare-nosed wombat range in NSW [51], the only confirmed localities in the state are Burrawang [46], Tooloom, Armidale [30] and Wee Jasper [17]. This study provides additional locality reports for *B. auruginans* and highlights the abundance of this species on bare-nosed wombats. Despite the host specificity of *B. auruginans*, it has been suggested that it is likely a three-host tick like other *Bothriocroton* sp., which parasitize reptiles [30]; however, further research on the life cycle and seasonality of this species is needed.

Known as the bush tick in Australia and the cattle tick or Asian longhorned tick elsewhere, *H. longicornis* is an introduced three-host tick distributed from south-east Queensland to Victoria [52]. A new tick record for bare-nosed wombats, the bush tick collected in this study was positively differentiated from similar species by 5+5 dentition and sharply pointed spurs on coxa 1 [30]. The adult male bush tick found in Coolagolite NSW is particularly unusual considering this species is an obligate parthenogen in Australia, resulting in males being quite rare [53]. Cattle, sheep and horses are the preferred hosts for this species, but it has also been collected from humans, domestic animals, various species of birds, black-striped wallabies (*Wallabia dorsalis*), northern brown bandicoots (*Isoodon macrourus*) and common wallaroos (*Macropus robustus*) [30, 31]. The three specimens collected in this study were from a free-ranging wombat on a 100-acre property with no active livestock; however, access to properties with livestock is possible across dried creek beds at certain times of the year (D. Ondinea, personal communication). The bush tick has been extensively studied overseas and is considered a vector of bacteria, viruses and protozoa, in particular *C. burnetii* [54], *Ehrlichia chaffeensis*, *Borrelia* spp. [55], and *Theileria orientalis* [56]; however, transmission has not been shown to occur in Australian specimens [57].

The Australian paralysis tick is well known for causing tick paralysis in domestic animals and humans [58]. Native Australian marsupials and eutherians have, however, co-evolved with the Australian paralysis tick, are the natural hosts for this tick and are typically immune to tick paralysis [59]. Found along the entire east coast of Australia, the Australian paralysis tick is an eclectic feeder and has been found on many different bird and mammal species; however, in certain areas it is dependent upon bandicoots to survive between seasons [30]. All specimens collected in this study were engorged females; however, adult males are rarely seen, as mating occurs off the host and adult male ticks feed on adult female ticks as opposed to the mammalian hosts [60]. With the use of targeted blocking primers a relapsing fever *Borrelia* sp. was recently isolated from a single Australian paralysis tick collected from an echidna [13], highlighting the hidden pathogenic potential of this species.

Like the Australian paralysis tick, the common marsupial tick is similarly indiscriminate in its feeding habits having been found on various wildlife, domestic animals and humans. However, it is the most widespread *Ixodes* species in Australia and has been associated with various pathogens such as *Rickettsia*, *Rickettsiella*, *Bartonella*, *Theileria*, nematodes and* Hepatozoon* [29, 61–66]. Regularly found on bare-nosed wombats in low numbers [48, 67], *I. tasmani* is a nidicolous species that detaches from its nocturnal vertebrate hosts during the day and is therefore likely associated with wombat burrows. Given its fast reproductive rate, three-host life cycle and the variety of pathogenic organisms that it typically harbours, this species is likely to pose a disease threat to wombats and wombat handlers; however, more research needs to be conducted to determine the extent of this threat.

An endemic tick that primarily feeds on macropods, wallaby tick is predominantly distributed throughout coastal Queensland and northern NSW, and apart from a disjunct population on Raymond Island Victoria, the southernmost reports of this species are from a bare-nosed wombat, a red-necked wallaby (*Macropus rufogriseus*) and a swamp wallaby (*Wallabia bicolor*) in the Nadgee State Forest NSW [24]. The specimens collected in the present study were from Dalgety and Quaama NSW, which are located approximately 2 h north of Nadgee. These new specimens further confirm the presence of wallaby tick in the far south NSW region and provide the second account of this species feeding on bare-nosed wombats [24]. Although macropods are the native host for *H. bancrofti*, there are more records of this species from cattle than native animals [24], and it is one of the main vectors of *T. orientalis* that impacts cattle in Australia [57, 68].

**Fig. 6:**
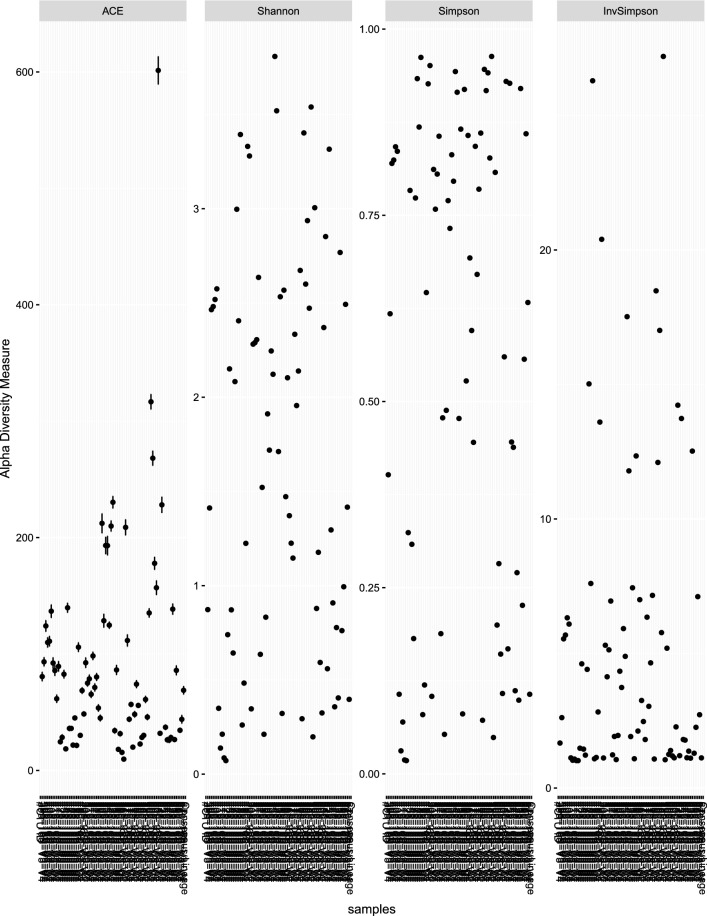
Alpha-diversity of bacterial composition in ticks collected from bare-nosed wombats (*V. ursinus*) between January 2019 and January 2020 assessed by diversity (Shannon, Simpson) and richness (ACE, inverse Simpson)

Analysis of alpha-diversity (Fig. 6) showed high variability across both sample locations and instars, similar to previous studies [17, 69]. However, there was some similarity between the same instars from the same collection location. Diversity can vary greatly between tick studies depending on extraction methods, the quality of filtering and bioinformatic analysis. All samples in this study underwent identical extraction, library preparation and bioinformatic analysis so it is possible that this affected sequencing depth of samples. It has been noted that for some species of native ticks, including the wombat tick, a larger number of sequences is required to produce an accurate representation of bacterial diversity [17]. The most abundant and diverse phylum was the Proteobacteria, which is consistent with similar studies of native hard ticks [29, 70].

Pathogens previously isolated from *B. auruginans* include *C. burnetii*, *Rickettsia massiliae* and *Rickettsia typhi* [29] and varying levels of Proteobacteria and Firmicutes [14, 17]. The very high prevalence of *C. burnetii* found in all *B. auruginans* instars in this study is similar to previous findings in this species [29]. *Coxiella*-like organisms are known to be highly efficient at transovarial transmission between tick hosts [71], and their presence within Malpighian tubules may suggest that they play a role in tick nutrition [7]. Different strains of *C. burnetii* have been shown to be highly related (> 99%) based on 16S rRNA, highlighting that the species recently evolved from an ancestral symbiont of ticks [6]. Because *B. auruginans* exhibits such remarkable host specificity, it is unlikely that this species is a significant vector for *C. burnetii* in humans. It is unknown, however, what impact this pathogen has on both healthy and sarcoptic mange-affected wombats. Blood and urine samples taken from wombats have failed to indicate the presence of *C. burnetii* [72], whereas other native marsupials such as the koala, bandicoots and macropods are regularly found to be seropositive for this bacterium [73–75]. Further studies to investigate the presence of *C. burnetii* in wombat faeces and blood, and in parasites other than *B. auruginans*, may be beneficial to determine the importance, role and impact of this pathogen in wombats and wombat ticks.

The presence of *Borrelia* in Australian ticks is a recent discovery [13], and targeted approaches using blocking primers and highly conserved housekeeping genes have provided insights into potential reservoirs and vectors of novel *Borrelia* sp. in Australia [14]. A species of *Borrelia* had the closest match to Candidatus *Borrelia ivorensis* and was detected in a single *Bothriocroton auruginans* from NSW. The original isolate for this species was from *Amblyomma variegatum* in western Africa, and it is more closely related to the relapsing fever *Borrelia* group than the Lyme group [76]. The uncultured *Anaplasma* sp. detected was originally isolated from an echidna tick (*Bothriocroton concolor*). All recognised *Anaplasma* spp. are obligate intracellular tick-borne mammalian pathogens [77], and as transovarial transmission between ticks has not yet been shown, it is believed that this genus persists solely through infected mammalian hosts [78].

A commensal bacterium of the mammalian gastrointestinal tract, *E. coli* is commonly found in native mammals, with the highest prevalence in herbivorous mammals with larger body masses [79]. Some species of *E. coli* are zoonotic and impact human health [80]. One study found northern hairy-nosed wombats (*Lasiorhinus krefftii*) to have an *E. coli* prevalence of 80% and southern hairy-nosed wombats (*Lasiorhinus latifrons*) to have 86% [79]; however, another study found no zoonotic *E. coli* in all three species of wombats [81]. While a strain of *E. coli* occurs in *B. auruginans*, it has been shown that ticks exhibit various innate immune responses to this bacterium [82, 83] and it is destroyed in the body of the tick rather than harboured and transmitted.

Ticks are often found to have large quantities of bacteria that are associated with the soil environments in which they spend most of their lives, in addition to bacteria associated with the skin of their mammalian hosts [13, 84]. Some pathogenic environmental and skin-associated bacteria that were detected in both the wombat tick and common marsupial tick may have potential implications for wombats with sarcoptic mange, or could even have been detected as a result of the ticks feeding on wombats with sarcoptic mange-associated bacteria.

Members of the genus *Staphylococcus* are typically commensals of mammalian skin, and are commonly found in ticks of native Australian wildlife [84, 85]; some species such as *Staphylococcus aureus* are associated with *Sarcoptes scabiei* mites and responsible for causing scabies-associated pyoderma in humans [86]. Two species of *Staphylococcus* were detected in this study: *S. agnetis*, which is typically associated with clinical disease in cattle and poultry [87, 88], and *S. sciuri*, which is a skin-associated bacterium acquired through contact with host skin [84]. *Staphylococcus sciuri* has also been detected in fleas from bandicoots and dogs in Australia, and in various lice and tick species including *I. holocyclus* and *H. longicornis* [85].

Further skin-related bacteria found included *C. ulcerans*, which causes a zoonotic infection similar to diphtheria [88], *Dolosigranulum pigrum*, which is associated with pneumonia in humans [89, 90], and *Macrococcus brunensis*, which is phylogenetically similar to a species of *Macrococcus* responsible for causing skin infection in dogs [91]. At least three distinct species of *Streptococcus* were detected, of which *S. dysgalactiae* and *S. didelphis* are important pathogens of humans and animals causing skin infection [92, 93]. Other species of *Streptococcus* such as *Streptococcus pyogenes* from *Sarcoptes scabiei* mites are responsible for causing skin infection in humans [86]. The pathogenicity and consequences of these skin-associated bacteria on both healthy and sarcoptic mange-impacted wombats may therefore be important.

Endosymbiotic bacteria are an important component of the tick microbiome and often play a role in tick reproductive and nutritional fitness [15]. The tick endosymbionts found in this study include *Rickettsiella*, *Acinetobacter* and *Pseudomonas*. The genera *Acinetobacter* and *Pseudomonas* have previously been isolated from wombat fleas [85]; they are also found in all *Ixodes* examined, and are believed to play an important role in the physiological processes of ticks [93]. Despite *I. tasmani* exhibiting a very high prevalence of a *Rickettsiella* endosymbiont, some known tick endosymbionts such as *Wolbachia* and *Francisella* were not detected in this study.

It is believed that bacterial endosymbionts are dominant in the majority of ixodid ticks [9], and there are examples of endosymbiotic bacteria so abundant they mask other microbes including pathogens, for example Candidatus *Midichloria mitochondrii* in the Australian paralysis tick [13]. DNA extracted from whole tick specimens, in particular those which have fed from their vertebrate hosts, will contain tick DNA, host DNA and microbial DNA (i.e. bacterial, viral, eukaryotic). The presence of host DNA in engorged ticks has been known to cause difficulties due to inhibitory properties in mammalian blood [94], so a targeted approach is required when examining bacterial communities. Popular genetic markers used for molecular identification of ticks and their associated bacteria, include the cytochrome* c* oxidase subunit 1 (COI) protein-coding gene, and the 16S rRNA, 12S rRNA and 18S rRNA genes [94]. Each has its advantages and limitations, for example COI offers an extensive existing library of universal primers as it is the standard marker for barcoding of animal species; however, it is limited in its ability to distinguish certain groups of organisms such as the Ixodidae to species level. The 16S rRNA gene is the most commonly used molecular marker because it can accurately distinguish between most prokaryotic taxa, but some microbial groups such as Rickettsiales may be difficult to distinguish due to their interspecific 16S rRNA similarity [95]. There are nine hypervariable regions of bacterial 16S rRNA genes that can be effectively targeted to identify bacterial taxa (V1-V9), and regions V1-V4 have been most commonly used in ticks [15].

The sampling method used in this study was both economical and allowed for a fair assessment of tick infestation rates on wombat hosts. However, it can be assumed that in some cases smaller nymphal and larval tick instars were likely overlooked. It is also likely that some ticks had left road-killed wombats which were opportunistically sampled, despite Skerratt et al. [48] finding no difference between tick density on live or road-killed wombats. The high abundance of female instars is likely indicative of some collection bias due to their larger size. The collection of ticks from animals in care limits the assessment of the origin of tick species and species of microorganisms due to the uncertainty of whether the ticks attached in the location of rehabilitation or the original habitat where the wombat was collected from. Three species of ticks collected from wombats (the wallaby, bush and Australian paralysis tick) could not be processed for bacterial presence in this study. However, these tick species are known vectors of significant pathogens of domestic animals and humans, and as a result have been extensively studied. All the ticks collected in this study except for the Australian paralysis tick were non-nidicolous hard ticks and presumably picked up by the wombat hosts whilst they were grazing. Many of the wombats used in this study were also in an atypical environment and had not had recent exposure to burrows. Considering that the majority of soft ticks are nidicolous and feed for very short periods of time, further investigation into the ticks associated with wombat burrows would provide a broader perspective of all the tick species associated with wombats.

## Conclusions

This study builds upon recent wildlife tick research and provides the first focused investigation into the ticks and tick-associated bacteria of bare-nosed wombats. The detection of various Proteobacteria in this study highlights the high bacterial diversity in native Australian ticks that was unrecognised prior to the development of NGS. Furthermore, the detection of *C. burnetii* in a large proportion of wombat ticks highlights the need for further investigation into wombat ectoparasites and their associated pathogens, in addition to the ability of wombats to cope with these pathogens and tick burdens in the presence of sarcoptic mange. The complex and dynamic relationships between vertebrate wildlife hosts, ticks and pathogens are continuously highlighted in the northern hemisphere [96, 97]. The unique evolutionary history of Australian fauna and tick species is shown in the distinct diversity yet taxonomic differences of these tick-host-pathogen relationships from those overseas. With the advancement of molecular methods the extent of these unique evolutionary relationships will become clearer, and may lead to potential improvements in the management of vector-borne diseases such as sarcoptic mange.

## Data Availability

The datasets used and/or analysed during the current study are available from the corresponding author on reasonable request. Voucher specimens have been submitted to the Australian Museum and include* Bothricroton auruginans* KS.130891,* Haemaphysalis bancrofti* KS.130892,* Ixodes holocyclus* KS.130893,* Ixodes holocyclus* KS.130893,* Haemaphysalis longicornis* KS.130894 and* Ixodes tasmani* KS.130895.

## Abbreviations

NGS: Next-generation sequencing
OTU: Operational taxonomic unit
COI: Cytochrome* c* oxidase subunit 1
